# Association between long-term healthy aging trajectories and all-cause mortality among middle-aged and older adults

**DOI:** 10.1186/s13690-025-01812-z

**Published:** 2025-12-22

**Authors:** Min Soo Kim, Hyun-Joo Kim, Jang Mook Kim

**Affiliations:** 1 Department of Public Health, General Graduate School of Dankook University, 119, Dandae-ro, Dongnam-gu, Cheonan-si, Chungcheongnam-do Republic of Korea; 2 Institute for Convergence Healthcare, Dankook University, 119, Dandae-ro, Dongnam-gu, Cheonan-si, Chungcheongnam-do Republic of Korea; 3 Department of Health & Medical Administration, Kyungmin University, 545, Seobu-ro, Uijeongbu-si, Gyeonggi-do Republic of Korea; 4 Department of Health Administration, College of Health Science, Dankook University, 119, Dandae-ro, Dongnam-gu, Cheonan-si, Chungcheongnam-do Republic of Korea

**Keywords:** Middle-aged and older adults, Healthy aging, All-cause mortality

## Abstract

**Background:**

Healthy aging is associated with lower mortality and better quality of life. This observational study examines how different healthy aging trajectories relate to all-cause mortality in middle-aged and older adults and seeks to identify subgroups at elevated risk based on individual characteristics.

**Methods:**

We used data from the Korean Longitudinal Study of Aging (KLoSA) collected between 2006 and 2020. The analytic sample comprised 4,168 adults aged 45 years and older. Healthy aging trajectories were derived from 2006 to 2014 using group-based trajectory modeling (GBTM) applied to a composite score (range 0–5) constructed from five binary domains: presence of chronic disease(s), limitations in activities of daily living (ADL), depressive symptoms, cognitive impairment (MMSE-K), and lack of social participation. Four trajectory groups were identified: sustained healthy aging, mild unhealthy aging, moderate unhealthy aging, and severe unhealthy aging. Associations between trajectory group and all-cause mortality during the 2014–2020 follow-up were estimated using Cox proportional hazards models; subgroup analyses were performed by gender and by age group.

**Results:**

Compared with the sustained healthy aging group, the moderate unhealthy aging group had higher all-cause mortality (HR = 1.946; 95% CI, 1.282–2.954; *p* = 0.002), and the severe unhealthy aging group also had higher mortality (HR = 2.222; 95% CI, 1.353–3.648; *p* = 0.002). Kaplan–Meier curves showed significant differences in survival across trajectory groups (log-rank *p* < 0.001). In subgroup analyses, females in the moderate (HR = 4.793; 95% CI, 1.833–12.534; *p* = 0.001) and severe (HR = 7.109; 95% CI, 2.592–19.498; *p* = 0.001) unhealthy aging groups had markedly higher mortality risk. Participants aged 45–64 years in the mild (HR = 2.493; 95% CI, 1.121–5.547; *p* = 0.025) and moderate (HR = 2.578; 95% CI, 1.092–6.082; *p* = 0.031) unhealthy aging groups also showed higher risk.

**Conclusion:**

Moderate and severe unhealthy aging trajectories were associated with higher all-cause mortality among middle-aged and older adults, particularly among females and adults aged 45–64 years. These findings underscore the need for tailored health management and support policies that account for gender and age differences.

**Supplementary Information:**

The online version contains supplementary material available at 10.1186/s13690-025-01812-z.

**Table:** 

Text box 1. Contributions to the literature
• This is the first nationally representative Korean analysis linking long-term, multidomain healthy aging trajectories (2006–2014) with subsequent seven-year all-cause mortality.
• The study models longitudinal trajectories rather than single time-point measures, enabling identification of individuals on worsening health paths.
• The analysis includes middle-aged adults (45–64 years), highlighting midlife as a practical window for prevention.
• The study uses a simple, non-clinical composite measure feasible for community or primary-care screening.
• It identifies high-risk subgroups by gender and age to guide targeted public-health strategies and resource allocation.

## Introduction

Population aging is accelerating worldwide due to improvements in medical care, technology, and living standards. In South Korea, the proportion of people aged 65 years and over exceeded 14% of the population in 2017 and reached approximately 18.4% by 2023, with projections indicating rapid further growth by 2050 [1, 2]. Aging increases the prevalence of multimorbidity and functional decline and raises the risks of social isolation and economic vulnerability, all of which impose growing demands on health and social care systems [3–7].

The World Health Organization defines healthy aging as “the process of developing and maintaining functional ability that enables well-being in older age,” highlighting multidimensional functioning rather than the mere absence of disease [8]. Prior research has linked healthy aging measures — including physical function, cognition, and selected biomarkers — to mortality and other adverse outcomes [9–15]. However, most existing studies have important limitations: they are frequently based on Western cohorts, often focus only on older adults (commonly ≥ 60 years), and typically use single time-point measures rather than characterizing long-term patterns of change [9–16]. Few nationally representative Asian studies have examined how long-term, multidomain aging patterns relate to mortality, and evidence on middle-aged adults (45–64 years) is especially scarce despite the policy relevance of earlier intervention.

In the Korean context, rapid demographic change, shifting family structures, and uneven regional resources shape both the distribution of aging trajectories and the feasibility of community-based interventions [17–19]. Although national policies (e.g., Health Plan initiatives and community care programs) aim to promote older adults’ health, many programs remain primarily health-service centric and may not sufficiently target social participation, mental health, or early prevention in midlife. Longitudinal trajectory evidence using nationally representative Korean data can therefore inform more precisely targeted prevention and resource allocation.

To address these gaps, we analyzed data from the Korean Longitudinal Study of Aging (KLoSA) to (1) identify long-term healthy aging trajectories using a five-domain composite measure and group-based trajectory modeling (GBTM) over 2006–2014, and (2) quantify the association between these trajectories and subsequent all-cause mortality during 2014–2020. Our study makes four key contributions: use of a multidomain, non-clinical healthy aging index; modeling of long-term trajectories rather than single time-point status; inclusion of middle-aged as well as older adults; and stratified analyses by gender and age group in a nationally representative Asian panel study. These features enhance the study’s policy relevance by identifying vulnerable subgroups who may benefit from earlier or more tailored interventions.

## Methods

### Data source and study subjects

**Fig. 1:**
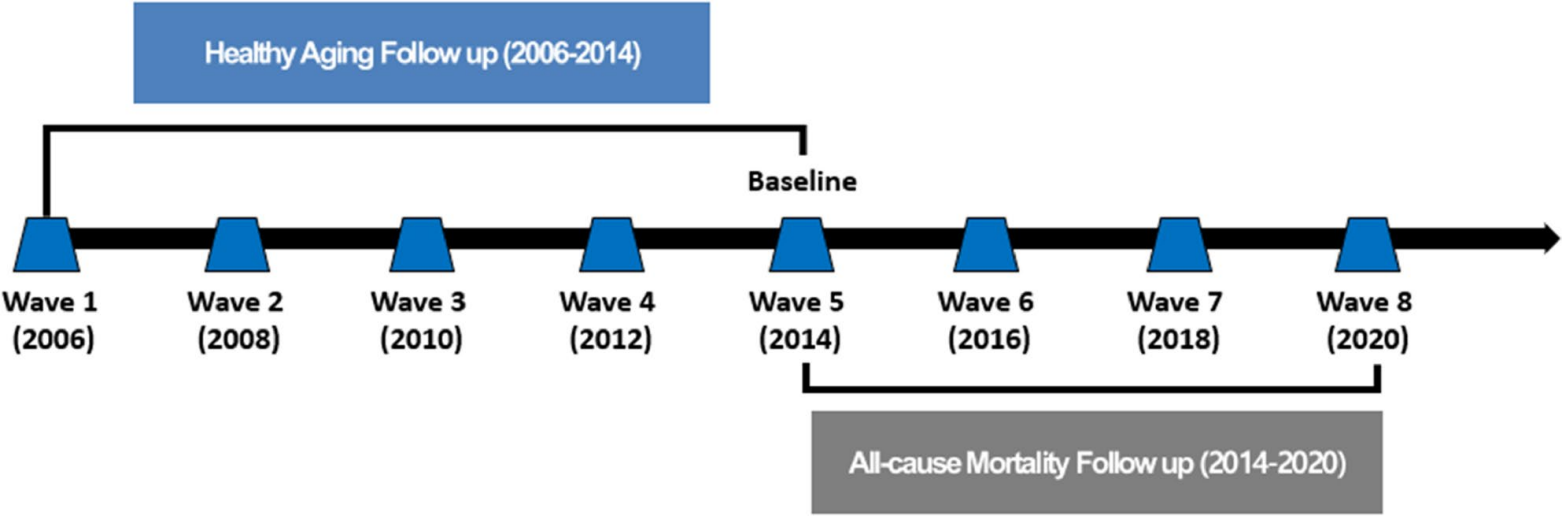
Tracking healthy aging trajectories (2006–2014) and subsequent all-cause mortality (2014–2020) among adults aged 45 years and older in Korea

**Fig. 2:**
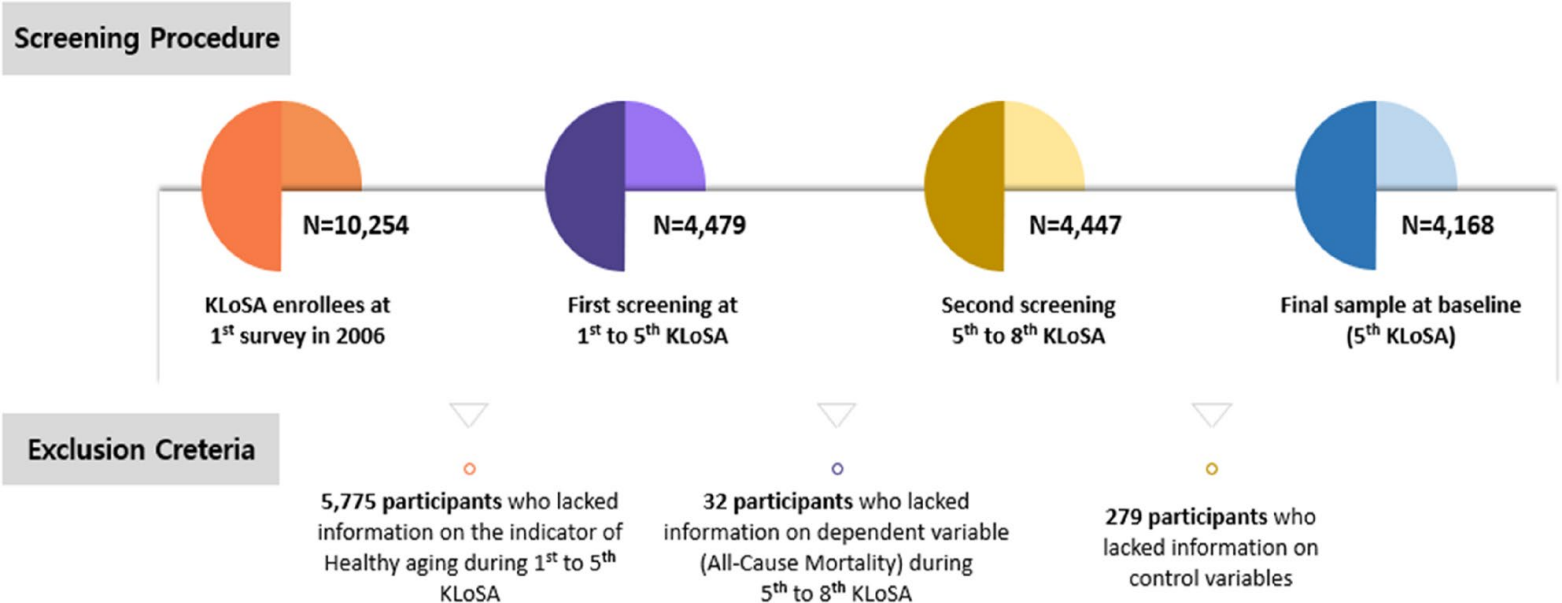
Flow diagram of participant selection from the Korean Longitudinal Study of Aging for the analytic sample (waves 1–8, 2006–2020)

This study used data from the Korean Longitudinal Study of Aging (KLoSA), administered by the Korea Employment Information Service, covering waves 1–8 (2006–2020). The study protocol was approved by the Institutional Review Board of Dankook University (IRB FILE No. 2024-10-024). KLoSA is a nationally representative biennial panel of individuals aged 45 years and older, designed to monitor social, economic, and health conditions in Korea [20]. For this analysis, we used waves 1–5 (2006–2014) to derive healthy aging trajectories and waves 5–8 (2014–2020) for mortality follow-up, with wave 5 (Aug 1, 2014) set as the baseline for survival analysis (Fig. 1). After applying exclusion criteria, the final analytic sample comprised 4,168 participants (Fig. 2).

### Independent variable: healthy aging

Healthy aging was operationalized as a composite of five binary domains: (1) self-reported chronic disease(s) (history of cancer, heart disease, chronic lung disease, diabetes, or cerebrovascular disease), (2) limitations in activities of daily living (ADL), (3) depressive symptoms (any of four screening items in the past week), (4) cognitive impairment (Mini-Mental State Examination (Korean version, MMSE-K) ≤ 23), and (5) lack of social participation. Each domain was coded 0/1 and summed (range 0–5), with higher scores indicating poorer healthy aging. These five domains were selected to capture the multidimensional construct of healthy aging (physical health, functional independence, mental health, cognition, and social engagement), consistent with prior work and policy frameworks [1, 8, 21] (Table S1).

### Dependent variable: all-cause mortality

All-cause mortality during 2014–2020 was ascertained from KLoSA exit interviews and linked mortality data. Time at risk was defined from the baseline interview (Aug 1, 2014) to the date of death, last contact, or end of follow-up (Dec 31, 2020), whichever occurred first. Participants without a recorded date of death by Dec 31, 2020 were censored at that date.

### Control variables

Control variables, selected a priori to control for confounding, comprised baseline sociodemographic, economic, lifestyle, and health-related characteristics: age (45–64, 65–74, ≥ 75), gender, educational level (≤ elementary, middle, high, ≥college), marital status (married vs. unmarried), residential area (metropolis/urban/rural), personal gross income (quintiles), current economic activity (employed vs. unemployed), current smoking and drinking, regular exercise (≥ 1 time/week), living alone, disability (physician diagnosed), subjective health status (good vs. bad), overall quality of life (0–100, categorized), and self-reported difficulty in daily activities due to vision or hearing impairment. Exact questionnaire items, response categories, and recoding rules are reported in Table S2 for transparency and reproducibility.

### Statistical analysis

**Fig. 3:**
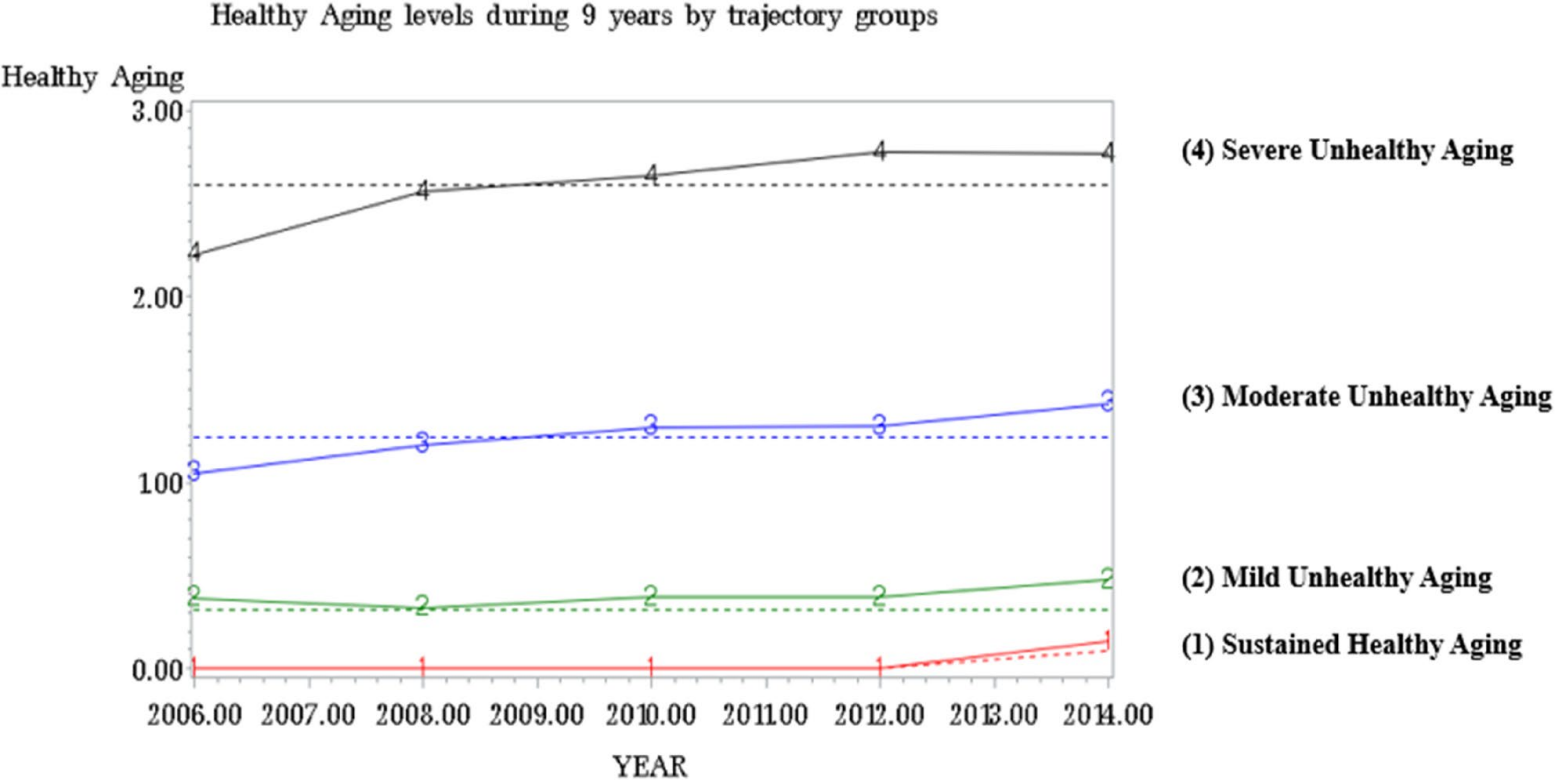
Healthy aging trajectories identified by group-based trajectory modeling among adults aged 45 years and older in Korea (2006–2014). KLoSA: Korean Longitudinal Study of Aging

The analysis employed a complete-case approach after excluding participants with missing values on independent, dependent, or control variables (exclusion counts are shown in Fig. 2). Sample characteristics were summarized with frequencies and percentages and compared using chi-square tests. Longitudinal healthy aging trajectories (2006–2014) were derived using group-based trajectory modeling (GBTM) implemented via PROC TRAJ in SAS. GBTM is a finite-mixture (latent-class) approach that estimates a set of group-specific polynomial trajectories to describe heterogeneity in longitudinal patterns and assigns individuals to the class with the highest posterior probability [22]. The CNORM (censored normal) specification was used to accommodate the bounded 0–5 composite healthy aging score. Candidate solutions with varying numbers of classes and polynomial orders (intercept, linear, quadratic, cubic) were compared using Bayesian Information Criterion (BIC), Average Posterior Probabilities (APP) (≥ 0.70), class sizes, and interpretability [23]; model selection statistics are reported in Table S3 and the selected 4-class solution is shown in Fig. 3. Associations between trajectory class and all-cause mortality were estimated using Cox proportional hazards models with robust standard errors; hazard ratios (HR) and 95% confidence intervals (CI) are reported. The proportional hazards assumption was evaluated graphically (log[− log] survival plots) and by Schoenfeld residual tests. In addition, the same covariates were included in stratified analyses for key variables such as gender and age, and additional sensitivity analyses were conducted using alternative age cut-offs (e.g., age ≥ 60 and ≥ 65). All statistical analyses were performed in SAS version 9.4 (SAS Institute Inc., Cary, NC).

## Results

### Trajectories of healthy aging

Figure 3 illustrates the four healthy aging trajectories identified among 4,168 participants over 2006–2014: sustained healthy aging (*n* = 1,089, 26.1%), mild unhealthy aging (*n* = 1,255, 30.1%), moderate unhealthy aging (*n* = 1,510, 36.2%), and severe unhealthy aging (*n* = 314, 7.5%).

### Sample characteristics and mortality distribution

Overall mortality during 2014–2020 was 8.5% (*n* = 355). The distribution of deaths across trajectory groups was: sustained healthy aging 8.5% (*n* = 30), mild unhealthy aging 17.7% (*n* = 63), moderate unhealthy aging 54.1% (*n* = 192), and severe unhealthy aging 19.7% (*n* = 70) (χ² = 176.7, *p* < 0.001) (Table 1).

**Table 1:** Baseline characteristics of study participants aged 45 years and older in Korea at wave 5 (*n* = 4,168)

Variables	Total		All-cause Mortality					
			Yes		No			
	*N*	%	*N*	%	*N*	%	χ2	*P*-value*
**Trajectory Healthy Aging**							176.7	< 0.001
Group 1: Sustained Healthy Aging	1,089	26.1	30	8.5	1,059	27.8		
Group 2: Mild Unhealthy Aging	1,255	30.1	63	17.7	1,192	31.3		
Group 3: Moderate Unhealthy Aging	1,510	36.2	192	54.1	1,318	34.6		
Group 4: Severe Unhealthy Aging	314	7.5	70	19.7	244	6.4		
**Age**							329.9	< 0.001
45–64	1,910	45.8	49	13.8	1,861	48.8		
65–74	1,355	32.5	99	27.9	1,256	32.9		
75-	903	21.7	207	58.3	696	18.3		
**Gender**							25.3	< 0.001
Male	1,629	39.1	183	51.5	1,446	37.9		
Female	2,539	60.9	172	48.5	2,367	62.1		
**Education Level**							89.1	< 0.001
≤ Elementary school	1,724	41.4	230	64.8	1,494	39.2		
Middle school	758	18.2	45	12.7	713	18.7		
High school	1,243	29.8	56	15.8	1,187	31.1		
≥ College	443	10.6	24	6.8	419	11.0		
**Marital Status**							57.2	< 0.001
Married	3,275	78.6	223	62.8	3,052	80.0		
Unmarried	893	21.4	132	37.2	761	20.0		
**Residential Area**							16.5	0.000
Metropolis	1,712	41.1	132	37.2	1,580	41.4		
Urban	1,385	33.2	100	28.2	1,285	33.7		
Rural	1,071	25.7	123	34.6	948	24.9		
**Personal Gross Income**							90.7	< 0.001
Quartile 1 (low)	812	19.5	51	14.4	761	20.0		
Quartile 2	812	19.5	95	26.8	717	18.8		
Quartile 3	851	20.4	127	35.8	724	19.0		
Quartile 4	909	21.8	49	13.8	860	22.6		
Quartile 5 (high)	784	18.8	33	9.3	751	19.7		
**Current Economic Activity**							47.3	< 0.001
Employed	1,764	42.3	89	25.1	1,675	43.9		
Unemployed	2,404	57.7	266	74.9	2,138	56.1		
**Current Smoking**							1.4	0.235
Yes	494	11.9	49	13.8	445	11.7		
No	3,674	88.1	306	86.2	3,368	88.3		
**Current Drinking**							18.3	< 0.001
Yes	1,415	33.9	84	23.7	1,331	34.9		
No	2,753	66.1	271	76.3	2,482	65.1		
**Regular Exercise**							14.7	0.000
Yes	1,468	35.2	92	25.9	1,376	36.1		
No	2,700	64.8	263	74.1	2,437	63.9		
**Living Alone**							13.8	0.000
Yes	545	13.1	69	19.4	476	12.5		
No	3,623	86.9	286	80.6	3,337	87.5		
**Disability**							4.9	0.026
Yes	17	0.4	4	1.1	13	0.3		
No	4,151	99.6	351	98.9	3,800	99.7		
**Subjective Health Status**							148.8	< 0.001
Good	3,219	77.2	182	51.3	3,037	79.6		
Bad	949	22.8	173	48.7	776	20.4		
**Overall Quality of Life**							70.7	< 0.001
Low	533	12.8	96	27.0	437	11.5		
High	3,635	87.2	259	73.0	3,376	88.5		
**Vision**							7.6	0.006
Yes	99	2.4	16	4.5	83	2.2		
No	4,069	97.6	339	95.5	3,730	97.8		
**Hearing**							57.8	< 0.001
Yes	87	2.1	27	7.6	60	1.6		
No	4,081	97.9	328	92.4	3,753	98.4		
**Total**	4,168	100.0	355	8.5	3,813	91.5		

^*^Chi-square tests

### Association between healthy aging trajectory and all-cause mortality

**Fig. 4:**
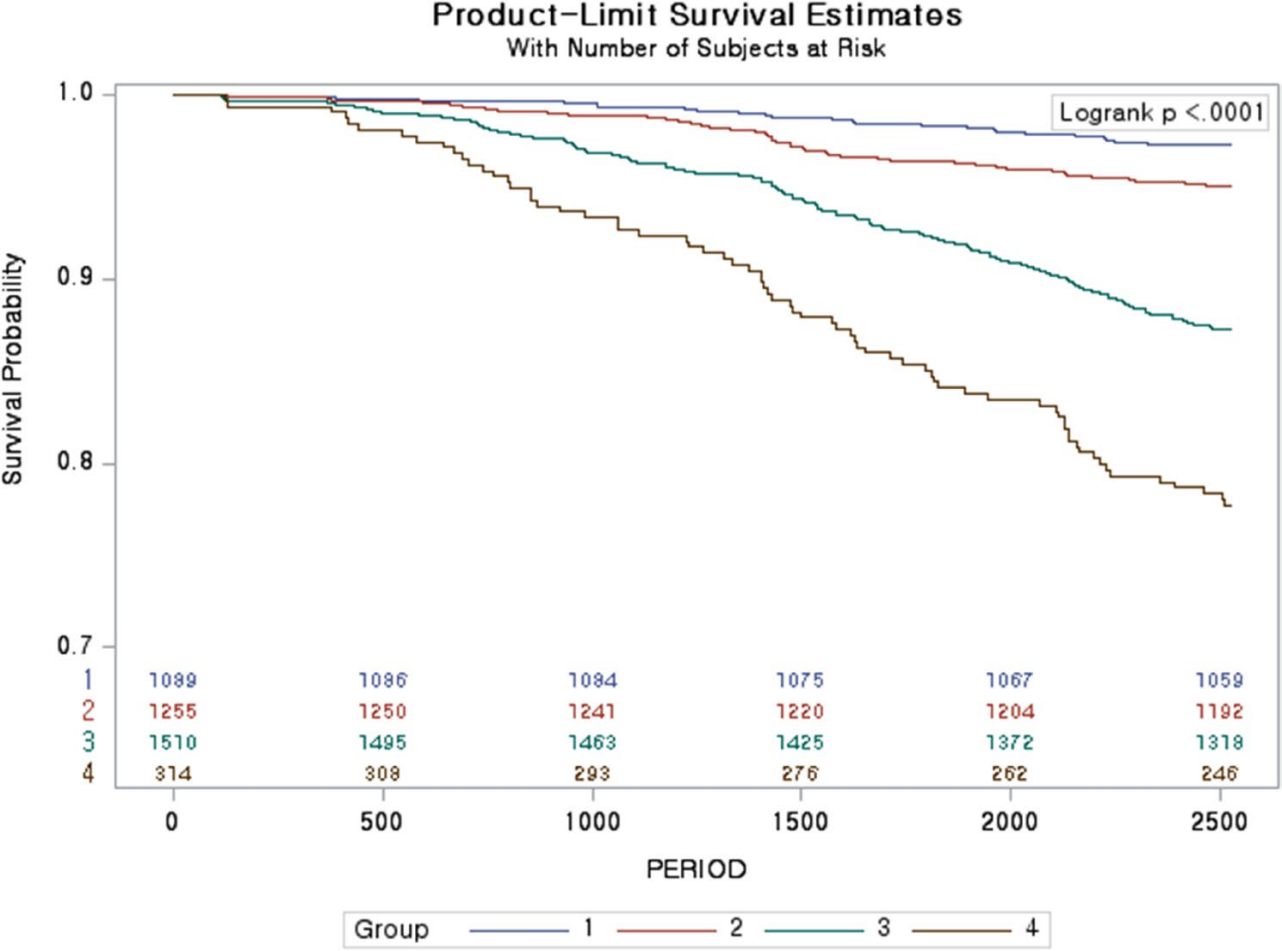
Kaplan–Meier plots of cumulative survival by healthy aging trajectory group among adults aged 45 years and older in Korea (baseline Aug 1, 2014; follow-up to Dec 31, 2020)

The proportional hazards assumption was assessed using log(− log[survival]) plots and Schoenfeld residual tests; no evidence of violation was observed. In multivariable Cox models adjusted for demographic and health covariates (Table 2), participants in the moderate unhealthy aging group had a higher risk of all-cause mortality compared with the sustained healthy aging group (HR = 1.946; 95% CI, 1.282–2.954; *p* = 0.002), and those in the severe unhealthy aging group also showed higher risk (HR = 2.222; 95% CI, 1.353–3.648; *p* = 0.002). Kaplan–Meier curves demonstrated significant differences in survival across trajectory groups (log-rank *p* < 0.001; Fig. 4). Overall, mortality risk increased progressively with worsening healthy aging trajectories.

**Table 2:** Association between healthy aging trajectory(2006–2014) and all-cause mortality (2014–2020) among adults aged 45 years andolder in Korea

Variables	All-cause Mortality				
	HR	95% CI			*P*-value
**Trajectory Healthy Aging**					
Group 1: Sustained Healthy Aging	1.000				
Group 2: Mild Unhealthy Aging	1.234	0.791	-	1.924	0.354
Group 3: Moderate Unhealthy Aging	1.946	1.282	-	2.954	0.002
Group 4: Severe Unhealthy Aging	2.222	1.353	-	3.648	0.002
**Age**					
45–64	1.000				
65–74	1.742	1.193	-	2.542	0.004
75-	3.738	2.539	-	5.503	< 0.001
**Gender**					
Male	3.562	2.703	-	4.695	< 0.001
Female	1.000				
**Education Level**					
≤ Elementary school	1.334	0.825	-	2.157	0.241
Middle school	1.065	0.637	-	1.782	0.809
High school	1.030	0.630	-	1.684	0.905
≥ College	1.000				
**Marital Status**					
Married	1.000				
Unmarried	1.918	1.410	-	2.607	< 0.001
**Residential Area**					
Metropolis	1.000				
Urban	0.803	0.616	-	1.047	0.106
Rural	1.039	0.797	-	1.354	0.777
**Personal Gross Income**					
Quartile 1 (low)	1.437	0.882	-	2.341	0.145
Quartile 2	1.179	0.745	-	1.864	0.483
Quartile 3	1.424	0.924	-	2.194	0.109
Quartile 4	0.913	0.575	-	1.450	0.701
Quartile 5 (high)	1.000				
**Current Economic Activity**					
Employed	1.000				
Unemployed	1.217	0.906	-	1.633	0.192
**Current Smoking**					
Yes	1.193	0.855	-	1.665	0.300
No	1.000				
**Current Drinking**					
Yes	0.629	0.478	-	0.827	0.001
No	1.000				
**Regular Exercise**					
Yes	1.000				
No	1.233	0.953	-	1.595	0.111
**Living Alone**					
Yes	0.634	0.450	-	0.892	0.009
No	1.000				
**Disability**					
Yes	0.862	0.313	-	2.377	0.775
No	1.000				
**Subjective Health Status**					
Good	1.000				
Bad	1.622	1.284	-	2.050	< 0.001
**Overall Quality of Life**					
Low	1.422	1.101	-	1.836	0.007
High	1.000				
**Vision**					
Yes	0.656	0.387	-	1.110	0.116
No	1.000				
**Hearing**					
Yes	1.552	1.025	-	2.350	0.038
No	1.000				

*HR* Hazard Ratio, *CI* Confidence Interval

### Stratified analyses by gender and age

Among female, the moderate (HR = 4.793; 95% CI, 1.833–12.534; *p* = 0.001) and severe (HR = 7.109; 95% CI, 2.592–19.498; *p* = 0.001) unhealthy aging groups exhibited markedly higher mortality risk relative to the sustained healthy aging group (Table 3). By age group, participants aged 45–64 years in the mild (HR = 2.493; 95% CI, 1.121–5.547; *p* = 0.025) and moderate (HR = 2.578; 95% CI, 1.092–6.082; *p* = 0.031) unhealthy aging groups showed elevated risk; among those aged ≥ 65 years, the moderate (HR = 1.738; 95% CI, 1.078–2.802; *p* = 0.023) and severe (HR = 2.125; 95% CI, 1.232–3.666; *p* = 0.007) groups were associated with higher mortality (Table 4).

**Table 3:** Association between healthy aging trajectory (2006–2014) and all-cause mortality (2014–2020), stratified by gender

**Variables**	** All-cause Mortality**				
	**Gender**				
	**Male**				
	**HR**	**95% CI**			***P*** **-value**
**Trajectory Healthy Aging**					
Group 1: Sustained Healthy Aging	1.000				
Group 2: Mild Unhealthy Aging	1.145	0.691	-	1.899	0.599
Group 3: Moderate Unhealthy Aging	1.500	0.934	-	2.410	0.094
Group 4: Severe Unhealthy Aging	0.815	0.375	-	1.769	0.605
**Variables**	**Female**				
	**HR**	**95% CI**			*P* **-value**
**Trajectory Healthy Aging**					
Group 1: Sustained Healthy Aging	1.000				
Group 2: Mild Unhealthy Aging	1.950	0.710	-	5.352	0.195
Group 3: Moderate Unhealthy Aging	4.793	1.833	-	12.534	0.001
Group 4: Severe Unhealthy Aging	7.109	2.592	-	19.498	0.000

*HR* Hazard Ratio, *CI* Confidence Interval

Adjusted age, education level, marital status, residential area, personal gross income, current economic activity, current smoking, current drinking, regular exercise, living alone, disability, subjective health status, overall quality of life, vision, hearing

### Sensitivity analyses by older age cut-offs

When using alternative age thresholds (age ≥ 60 and age ≥ 65) (Table 5), the moderate and severe unhealthy aging groups remained associated with higher mortality among those aged ≥ 60 (HR = 1.760; 95% CI, 1.134–2.732; *p* = 0.012 and HR = 1.937; 95% CI, 1.157–3.243; *p* = 0.012, respectively), whereas at age ≥ 65 only the severe group remained significantly associated with higher risk (HR = 1.824; 95% CI, 1.053–3.159; *p* = 0.032).

**Table 4:** Association between healthy aging trajectory (2006–2014) and all-cause mortality (2014–2020), stratified by age group

**Variables**	**All-cause Mortality**				
	**Age**				
	**45–64**				
	**HR**	**95% CI**			***P*** **-value**
**Trajectory Healthy Aging**					
Group 1: Sustained Healthy Aging	1.000				
Group 2: Mild Unhealthy Aging	2.493	1.121	-	5.547	0.025
Group 3: Moderate Unhealthy Aging	2.578	1.092	-	6.082	0.031
Group 4: Severe Unhealthy Aging	1.290	0.142	-	11.741	0.821
**Variables**	**≤ 65**				
	**HR**	**95% CI**			***P*** **-value**
**Trajectory Healthy Aging**					
Group 1: Sustained Healthy Aging	1.000				
Group 2: Mild Unhealthy Aging	0.938	0.551	-	1.596	0.813
Group 3: Moderate Unhealthy Aging	1.738	1.078	-	2.802	0.023
Group 4: Severe Unhealthy Aging	2.125	1.232	-	3.666	0.007

*HR* Hazard Ratio, *CI* Confidence Interval

Adjusted gender, education level, marital Status, residential area, personal gross income, current economic activity, current smoking, current drinking, regular exercise, living alone, disability, subjective health status, overall quality of life, vision, hearing

**Table 5:** Sensitivity analyses of associations between healthy aging trajectories and all-cause mortality using alternative age cut-offs among Korean adults

**Variables**	**All-cause Mortality**				
	**60-**				
	**HR**	**95% CI**			***P*** **-value**
**Trajectory Healthy Aging**					
Group 1: Sustained Healthy Aging	1.000				
Group 2: Mild Unhealthy Aging	1.011	0.624	-	1.637	0.965
Group 3: Moderate Unhealthy Aging	1.760	1.134	-	2.732	0.012
Group 4: Severe Unhealthy Aging	1.937	1.157	-	3.243	0.012
**Variables**	**65-**				
	**HR**	**95% CI**			***P*** **-value**
**Trajectory Healthy Aging**					
Group 1: Sustained Healthy Aging	1.000				
Group 2: Mild Unhealthy Aging	0.873	0.513	-	1.488	0.618
Group 3: Moderate Unhealthy Aging	1.604	0.994	-	2.590	0.053
Group 4: Severe Unhealthy Aging	1.824	1.053	-	3.159	0.032

*HR* Hazard Ratio, *CI* Confidence Interval

Adjusted age, education level, marital status, residential area, personal gross income, current economic activity, current smoking, current drinking, regular exercise, living alone, disability, subjective health status, overall quality of life, vision, hearing

## Discussion

Long-term trajectories of healthy aging were strongly associated with subsequent all-cause mortality. Compared with the sustained healthy aging trajectory, membership in the moderate and severe unhealthy aging trajectories was associated with substantially higher mortality after adjustment for baseline covariates; these associations were most pronounced among female and among adults aged 45–64 years. By modeling multidomain healthy aging longitudinally and including middle-aged as well as older adults in a Korean sample, the study extends prior work that has largely relied on single time-point measures or Western cohorts [9–11, 13–15, 24].

Our results indicate a graded relationship between worsening aging trajectories and mortality risk: participants whose composite scores worsened or remained poor over the 2006–2014 period experienced higher hazards of death during 2014–2020. This pattern plausibly reflects cumulative loss of physiological reserve and functional capacity (physical, cognitive, mood, and social domains), increasing vulnerability to acute illness, complications, and fatal outcomes [25–31]. The stronger associations observed among female may reflect an interaction of biological and social factors — such as postmenopausal hormonal changes, differing comorbidity and disability patterns, and socioeconomic or healthcare access disparities — that amplify the downstream mortality impact of long-standing deficits [32–36]. Importantly, our finding that adverse trajectories in the 45–64 age group predict mortality underscores the relevance of midlife health patterns: declines that begin or consolidate in midlife may accelerate risk later, suggesting a window for earlier detection and intervention [37–42].

Compared with previous studies using clinical biomarkers or single-domain measures, our use of a non-clinical, multidomain composite has practical advantages for community surveillance and primary-care screening because it relies on feasible assessments (self-report, brief cognition, ADL, and social participation) that nonetheless capture heterogeneity relevant for mortality prediction [9, 10, 13–15]. The identification of trajectory-defined subgroups provides actionable stratification: it highlights not only who has worse health at one time point but who is on a worsening path, which has different implications for prevention and resource allocation.

These patterns are also likely to be reinforced by interactions among multimorbidity, functional decline, and treatment complexity. Individuals with multiple chronic conditions commonly face polypharmacy and higher care complexity, which may reduce physiological resilience and increase vulnerability to infection and adverse outcomes [31, 43, 44]. Mental health problems such as depression and anxiety often co-occur with functional decline and may impair healthcare utilization or adherence, contributing to higher mortality risk [1, 21, 45–48]. We emphasize that our findings are observational and do not by themselves prove that intervening on healthy aging trajectories will reduce mortality. Nevertheless, the temporal ordering, the graded associations across trajectory severity, and the robustness in sensitivity analyses increase the plausibility that trajectory-defined risk stratification can be useful for population surveillance and early risk identification; establishing that specific interventions reduce mortality, however, requires studies designed for causal inference (e.g., randomized trials or longitudinal causal methods) [49].

### Clinical implications

These findings support integrating simple, multidomain aging assessments into routine primary-care and community screening to identify individuals on adverse trajectories. For middle-aged adults, such screening could trigger targeted risk-factor management, lifestyle interventions, and psychosocial supports aimed at halting or reversing decline. For older adults, interventions should prioritize maintaining function and managing multimorbidity through integrated care, rehabilitation, and social support. Policy measures that expand access to community-based programs combining physical activity, mental-health services, and social engagement may help mitigate trajectory-related risk at the population level [17–19, 50].

### Limitations, strengths, and future studies

This study has limitations. First, several healthy aging domains and covariates were self-reported, which may introduce measurement error and reporting bias. Second, institutionalized individuals were not included, possibly overestimating population-level healthy aging. Third, the observational design precludes definitive causal inference: residual confounding, measurement error, and potential reverse causation remain possible despite multivariable adjustment. We therefore interpret associations cautiously and note that future analyses applying formal causal methods (e.g., marginal structural models, g-computation, targeted maximum likelihood estimation, or instrumental-variable approaches) and quantitative sensitivity analyses (such as E-values) would be required to better estimate causal effects of interventions. Fourth, the primary analysis used a complete-case approach; we report exclusion counts (Fig. 2) and age-stratified sensitivity checks, but additional robustness checks would further assess potential bias. Finally, mortality ascertainment relied on exit interviews and linked data, which may not be fully concordant with death certificates.

Strengths include the long follow-up (14 years across exposure and outcome windows), use of a large, nationally representative sample, multidomain longitudinal measurement of healthy aging, and rigorous trajectory modelling. Future research should validate these trajectory classes against clinical biomarkers and objective physical measures, apply causal inference methods to better estimate intervention effects, and test targeted interventions in midlife to determine whether altering adverse trajectories reduces mortality.

## Conclusion

Moderate and severe unhealthy aging trajectories were associated with higher all-cause mortality among middle-aged and older adults, with particularly pronounced associations among female and among adults aged 45–64 years. These results support the value of population-level surveillance of multidomain aging indicators and the development of age- and gender-tailored strategies for risk identification and prioritization of preventive resources, while acknowledging that causal effects of specific interventions remain to be established.

## Supplementary Information

Supplementary Material 1.

## Data Availability

The study used data from the Korean Longitudinal Study of Aging (KLoSA). The data are available from the Korea Employment Information Service (KEIS) and can be accessed via the KLoSA data portal: [https://survey.keis.or.kr/klosa/klosa04.jsp] (https:/survey.keis.or.kr/klosa/klosa04.jsp) . The authors are not authorized to share the dataset directly. Researchers wishing to use these data should request access through the KEIS/KLoSA website and comply with the data-use agreement and application procedures specified there.

## Abbreviations

ADL: Activities of Daily Living
AIC: Akaike Information Criterion
APP: Average Posterior Probability
BIC: Bayesian Information Criterion
CNORM: Censored Normal
GBTM: Group Based Trajectory Modeling
HR: Hazard Ratio
KLoSA: Korean Longitudinal Study of Aging
LL: Log-Likelihood
MMSE-K: Mini Mental State Examination-Korean version
PH: Proportional Hazards
CI: Confidence Interval
