# Supplementary material for: Association between long-term healthy aging trajectories and all-cause mortality among middle-aged and older adults
Source: Arch Public Health. 2025 Dec 22;84:17. doi: 10.1186/s13690-025-01812-z (PMC12837901; doi:10.1186/s13690-025-01812-z)
Supplement: Supplementary file 1 — Supplementary Material 1. [file 13690_2025_1812_MOESM1_ESM.docx]

Table S1: Components and coding rules for the healthy aging composite measure (2006–2014)

| **Domain** | **Item / question** | **Coding (analysis)** | **Notes** |
| --- | --- | --- | --- |
| Chronic disease status | “Have you ever been diagnosed by a doctor with any of the following: cancer, heart disease, chronic lung disease, diabetes, cerebrovascular disease?” | **0** = no diagnosis of any listed disease  **1** = diagnosed with ≥1 listed disease | Self-reported physician diagnosis. Presence of any of the five diseases yields 1 point. |
| ADL limitation (disability) | Assistance required for any ADL: bathing, eating, dressing, walking across a room, getting in/out of bed, or using the bathroom | **0** = no assistance needed for any ADL  **1** = needs assistance with ≥1 ADL | Based on standard ADL items. Any reported assistance → 1 point. |
| Depressive symptoms | During the past week, have you often felt: (a) sad; (b) lonely; (c) depressed; (d) that everything you did was an effort? (yes/no for each) | **0** = none of the four items endorsed **1** = any item endorsed | Brief 4-item screening derived from KLoSA questionnaire. Any positive response counted as depressive-symptom flag. |
| Cognitive impairment (MMSE‑K) | Mini-Mental State Examination—Korean version (MMSE‑K) total score | **0** = MMSE‑K ≥ 24 (normal) **1** = MMSE‑K ≤ 23 (impairment) | Cutoff consistent with standard MMSE‑K thresholds used in KLoSA analyses. |
| Social participation | Participation in any of: religious gatherings, social gatherings, leisure/culture/sports organizations, alumni/hometown/family associations, volunteer work, political/civic/interest groups, or other | **0** = participates in ≥1 activity **1** = participates in none | Non-participation indicates social isolation / low social engagement → 1 point. |

ADL: Activities of Daily Living; MMSE-K: Mini Mental State Examination-Korean version; KLoSA: Korean Longitudinal Study of Aging.

Table S2: Definitions and coding of control variables used in survival models

| **Variable** | **Source / Definition** | **Coding (analysis)** | **Notes** |
| --- | --- | --- | --- |
| Age | Age in years at baseline (wave 5, 2014) | 45–64 / 65–74 / ≥75 | Recorded as years |
| Gender | Self-reported gender | Male / Female | Coded as binary |
| Education level | Highest completed schooling | ≤ Elementary school / Middle school / High school / ≥ College | See KLoSA item for exact response options |
| Marital status | Current marital status | Married / Unmarried | Unmarried = separated/divorced/widowed/never; Respondents currently married coded as Married |
| Residential area | Administrative residence | Metropolis / Urban / Rural | Metropolis = Seoul and other major metropolitan cities / Urban = other cities / Rural = county-level areas |
| Personal gross income | Sum of earned income, asset income, public transfers, financial support, pension, other income | Quintiles (1 = lowest … 5 = highest) | Income computed on annual basis; quintiles calculated across analytic sample |
| Current economic activity | Self-reported current work status | Employed / Unemployed | Employed = currently working (paid job or self-employment) / Unemployed = currently not working (unemployed or economically inactive) |
| Current smoking | Question: "Do you currently smoke?" | Yes / No | Yes=Current smoker / No=Non-smoker or former smoker |
| Current drinking | Self-reported drinking frequency | Yes / No | Yes = Current drinker(Current drinker includes occasional or frequent drinkers or recent consumption) / No = Non-drinker or former drinker |
| Regular exercise | Question: "Do you exercise at least once a week?" | Yes / No | Binary indicator of weekly exercise |
| Living alone | Household composition | Yes / No | Living alone defined as household size = 1 |
| Disability | Physician-diagnosed disability | Yes / No | Based on self-report of physician diagnosis |
| Subjective health status | Self-rated health | Good / Bad | Good (very good / somewhat good / average) / Bad (somewhat bad / very bad); Collapsed into two categories for models |
| Overall quality of life (QoL) | Visual scale 0–100 (10-point increments) | Low / High | Low (≤40) / High (≥50);  Original responses recorded in 10-point increments |
| Difficulty due to vision | Self-report of difficulty in daily activities because of vision | Yes / No | Coded as binary |
| Difficulty due to hearing | Self-report of difficulty in daily activities because of hearing | Yes / No | Coded as binary |

KLoSA: Korean Longitudinal Study of Aging.

Table S3: Model selection and evaluation statistics for group-based trajectory modeling of healthy aging (2006–2014)

| **Categories** | | **1 Class** | | **2 class** | | **3 class** | | **4 class** | |
| --- | --- | --- | --- | --- | --- | --- | --- | --- | --- |
| Model fit index | BIC^2 a^ | -27937.24 | | -24300.6 | | -23682.1 | | -23552.3 | |
|  | BIC^3 b^ | -27939.65 | | -24305.4 | | -23689.4 | | -23560.3 | |
|  | AIC | -27927.74 | | -24281.6 | | -23653.6 | | -23520.6 | |
|  | LL | -27924.74 | | -24275.6 | | -23644.6 | | -23510.6 | |
| Sub-group (%) and Average posterior probability | 1 Class | 100.0 | 100.00 | 56.2 | 0.94 | 47.3 | 0.94 | 26.1 | 0.86 |
|  | 2 Class |  |  | 43.8 | 0.98 | 42.4 | 0.92 | 30.1 | 0.91 |
|  | 3 Class |  |  |  |  | 10.3 | 0.91 | 36.2 | 0.89 |
|  | 4 Class |  |  |  |  |  |  | 7.5 | 0.89 |
| AIC: Akaike Information Criterion; LL: Log-Likelihood.  ^a^: Bayesian information criterion (for the total number of participants)/N = 4,168 ^b^: Bayesian information criterion (for the total number of observations)/N = 20,840 | | | | | | | | | |
